# A non-syndromic intellectual disability associated with a *de novo* microdeletion at 7q and 18p, microduplication at Xp, and 18q partial trisomy detected using chromosomal microarray analysis approach

**DOI:** 10.1186/1755-8166-7-44

**Published:** 2014-06-27

**Authors:** Irene Plaza Pinto, Lysa Bernardes Minasi, Alex Silva da Cruz, Aldaires Vieira de Melo, Damiana Míriam da Cruz e Cunha, Rodrigo Roncato Pereira, Cristiano Luiz Ribeiro, Claudio Carlos da Silva, Daniela de Melo e Silva, Aparecido Divino da Cruz

**Affiliations:** 1 Departamento de Biologia, Pontifícia Universidade Católica de Goiás, Núcleo de Pesquisas Replicon, Rua 235, n. 40, Bloco L, Área IV Setor Universitário, Goiânia, GO, Brazil; 2 Universidade Federal de Goiás, Instituto de Ciências Biológicas, Programa de Pós Graduação em Biologia, Campus Samambaia, Goiânia, GO, Brazil; 3 Departamento de Biologia, Pontifícia Universidade Católica de Goiás, Mestrado em Genética, Programa de Pós Graduação Mestrado em Genética, Rua 235, n. 40, Bloco L, Área IV Setor Universitário, Goiânia, GO, Brazil; 4 Universidade Federal de Goiás, Programa de Pós Graduação em Biotecnologia e Biodiversidade, Rede Centro Oeste de Pós Graduação, Pesquisa e Inovação, Campus Samambaia, Goiânia, GO, Brazil; 5 Programa de Pós-Graduação em Genética e Biologia Molecular, Laboratório de Genética e Biodiversidade, Universidade Federal de Goiás, Goiânia, GO, Brazil; 6 Laboratório de Citogenética Humana e Genética Molecular, Secretaria do Estado da Saúde de Goiás (LACEN/SESGO), Goiânia, GO, Brazil

**Keywords:** Intellectual disability, CMA, 18q partial trisomy, Microdeletion, Microduplication, Mosaicism

## Abstract

**Background:**

Chromosome abnormalities that segregate with a disease phenotype can facilitate the identification of disease loci and genes. The relationship between chromosome 18 anomalies with severe intellectual disability has attracted the attention of cytogeneticists worldwide. Duplications of the X chromosome can cause intellectual disability in females with variable phenotypic effects, due in part to variations in X-inactivation patterns. Additionally, deletions of the 7qter region are associated with a range of phenotypes.

**Results:**

We report the first case of *de novo* microdeletion at 7q and 18p, 18q partial trisomy, microduplication at Xp associated to intellectual disability in a Brazilian child, presenting a normal karyotype. Karyotyping showed any chromosome alteration. Chromosomal microarray analysis detected a *de novo* microdeletion at 18p11.32 and 18q partial trisomy, an inherited microdeletion at 7q31.1 and a *de novo* microduplication at Xp22.33p21.3.

**Conclusions:**

Our report illustrates a case that presents complex genomic imbalances which may contribute to a severe clinical phenotypes. The rare and complex phenotypes have to be investigated to define the subsets and allow the phenotypes classification.

## Background

Chromosome abnormalities that segregate with a disease phenotype can facilitate the identification of disease loci and genes. The relationship between chromosome 18 anomalies with severe intellectual disability (ID) has attracted the attention of cytogeneticists worldwide [1]. Moreover, the partial trisomy of the chromosome 18 is a rare genetic chromosomal syndrome, corresponding to a variant and less severe form of Edwards’s Syndrome (OMIM 300484). For this condition, the severity of the symptoms and the phenotype is highly variable depending on the extension of chromosome involvement and the level of compromised cells and tissues [2].

Eighty percent of the cases of Edwards’s Syndrome present full trisomy, the other 20% present mosaic or partial 18 trisomy [1]. After birth, mosaic chromosomal abnormalities are essentially identified among individuals with phenotypic manifestation of recognizable aneuploidy (chromosomal) syndromes. Mosaic aneuploidy involving 18 chromosome was classified as a frequent mosaic autosomal aneuploidy [3].

Several genomic disorders have been linked to X chromosome. Duplications of the X chromosome can cause ID in females with variable phenotypic effects, due in part to variations in X-inactivation patterns [4]. It has been estimated that up to 30% of genes on the short arm of the X chromosome could escape inactivation and undergo gene expression in cells and tissues [5]. Deletions of the 7qter region are associated with a range of phenotypes, and clinical findings comprise low birth weight, mental retardation, development delay, facial dysmorphisms and genitourinary malformations [6,7].

Here in we report the first case of *de novo* microdeletion at 7q and 18p, 18q partial trisomy, microduplication at Xp associated to a non-syndromic intellectual disability in a Brazilian child, presenting a normal karyotype.

## Case presentation

A 4-years old female patient born to non-consanguineous parents, at 39 weeks gestation to a 33-year-old mother and 47-year-old father, and her birth weight was 2325 g. Child delivery was carried out through a caesarean section procedure. Physical examination of the proband reveled delayed psychomotor development, severe intellectual disability, and a height of 94,5 cm (<7rd), a weight of 9500 g (<7rd). Her craniofacial dysmorphisms included an oblong face with a prominent forehead/frontal bossing, a bulbous nasal tip on a small nose and abnormal teeth. She also had hypertelorism, lower ear implantation and limb weakness. The family history was unremarkable on both sides.

### Results

**Figure 1:**
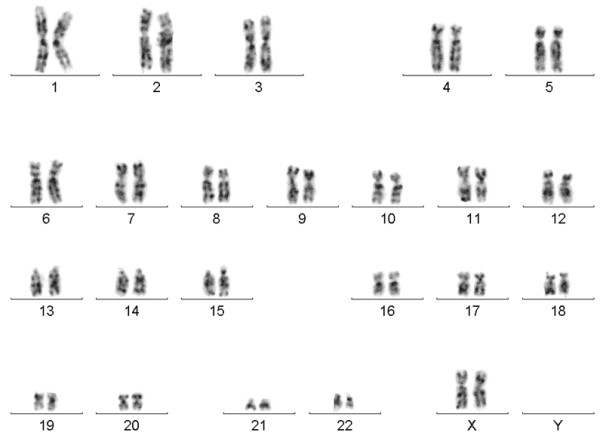
**G-banded karyotype.** Displaying no numerical or structural karyotype deviations (46,XX).

**Figure 2:**
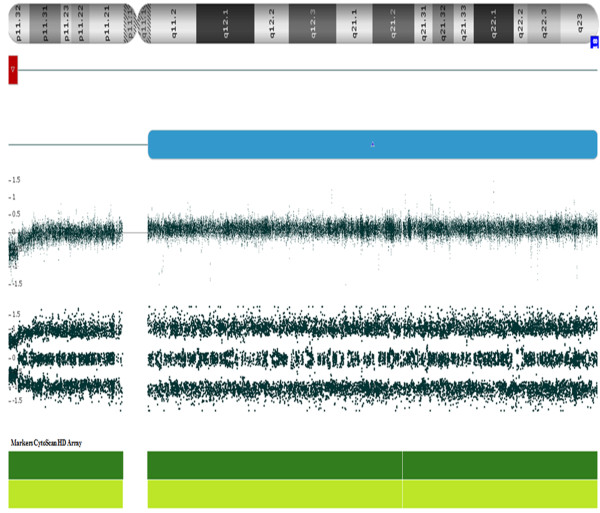
**CMA depicts genomic imbalances in chromosome 18.** The microarray profile of the 18p11.32 microdeletion is represented by the bold red line and the 18q partial trisomy is represented by the bold blue line, both in mosaicism.

**Figure 3:**
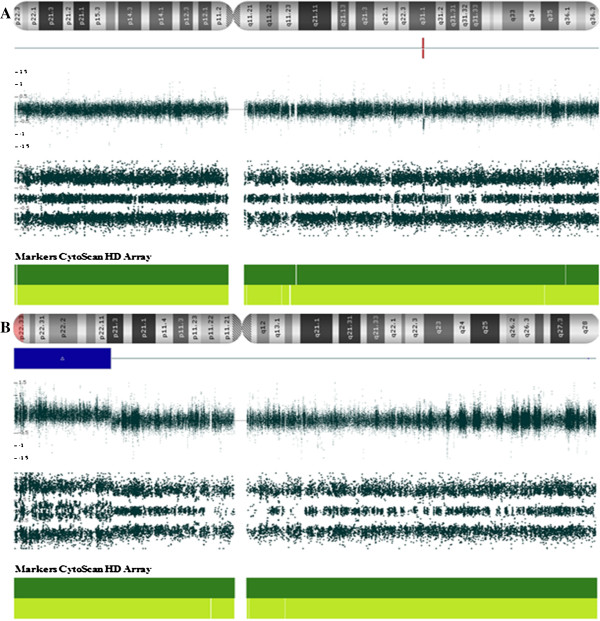
**CMA data of the proband with genomic imbalances in 7 and X chromosomes.** Analysis showing **(A)** a deletion segment at 7q31.1 and **(B)** duplication segment at Xp22.33p21.3 from patient.

Karyotyping at about 550 band resolution showed the proband with a female karyotype (46,XX), without any suggestion of chromosome alteration (Figure 1). Her parents also presented normal karyotype. CMA detected four genomic imbalances in the patient’s genome, corresponding to a *de novo* 1.23 Mb microdeletion at 18p11.32(136,226-1,369,804)x1 [NCBI 37.3/hg19] with 30% mosaicism, a 18q partial trisomy with 40% mosaicism (Figure 2), an inherited 386.73 kbp microdeletion at 7q31.1(110,923,434-111,310,159)x1 [NCBI 37.3/hg19] and a *de novo* 25.72 Mb microduplication at Xp22.33p21.3(168,546-25,887,307)x3 [NCBI 37.3/hg19] (Figure 3). The progenitor’s CMA confirmed *de novo* genomic imbalances in their child (Table 1).

**Table 1:** Clinical and molecular features of patient

Clinical features*	**Age (yo)**	**Sex**	**CNV**	**Mosaic (%)**	**Cytoband**	**Size (Mb)**	**Marker count**	**Microarray nomenclature**	**Number of genes**	Selected OMIN Morbid Genes**	**Origin**	**Interpretation**
GDD, MS, MCA	4	F	Loss		7q31.1	0.39	265	7q31.1(110,923,434-111,310,159)x1	1	*IMMP2L*	Inherited mat	LP
			Loss	30	18p11.32	1.23	1400	18p11.32(136,226-1,369,804)x1	11	*ADCYAP1*	*de novo*	LP
			Gain	40	18q11.1		53197	18q11.1q23(18,608,373-78,014,123)*x*2-3		*18q Partial Trissomy*	*de novo*	Pathogenic
			Gain		Xp22.33	25.72	31456	Xp22.33p21.3(168,546-25,887,307)x3	147	*NLGN4X, AP1S2, NHS, CDKL5, RPS6KA3, MBTPS2, SMS, ARX*	*de novo*	Pathogenic

*GDD = Global Developmental Delay; MS = Multiple Stigmas, MCA = Multiple Congential Anomalies; **Genes related to ID/Autsm; yo = years old; LP = Likely Pathogenic.

### Discussion

In our study, the use of microarray analyses allowed the identification of genomic rearrangements in a girl severely affected with intellectual disability, multiple congenital abnormalities and intense dysmorphology, despite her normal karyotype.

CMA showed 386.73 kbp microdeletion at 7q31.1, inherited from her mother, likely pathogenic because harbor *IMMP2L* gene that, according to authors, deletions involving this gene have been associated with attention deficit hyperactivity disorder (ADHD) [8], autism [9], and Tourette syndrome [10]. Moreover, the *IMMP2L* gene was also found in inherited rare CNV-associated gene set in ADHD patients [8].

Furthermore, we observed two genomic imbalances at 18 chromosome. A *de novo* mosaic microdeletion at 18p11.32, a genomic imbalance comprising 11 genes, and the gene only was *ADCYAP1*, also known as *PACAP*, related to intellectual disability [11]. Additionally, the monosomy of 18p chromosome refers to a chromosomal disorder, resulting from the absence of all or part of the short arm, and the clinical features frequently include mild to moderate mental retardation, short stature, and speech delay [12].

On the other hand, the most relevant alteration observed was a *de novo* 18q partial trisomy with 40% mosaicism. The degree of clinical manifestations in a patient who presents partial trisomy of chromosome 18 is variable, generally include a relatively mild phenotype with a high survival rate [1]. In the partial trisomy form, only a segment of the chromosome 18q is present in triplicate, often resulting from a balanced translocation or an inversion carried by one parent. This type of trisomy accounts for approximately 2% of cases presenting with the Edward’s phenotype. The location and the extent of the triplicated segment and the possible associated deletion of genomic material due to unbalanced translocation can explain the variable phenotype associated with partial trisomies [13].

The clinical findings are highly variable in a phenotypic spectrum that spans from the absence of dysmorphic features with normal intelligence to the complete trisomy 18 syndrome. Thus, clinical and cytogenetic diagnostic of chromosome 18 partial trisomy in mosaic state is a challenge for both medical and laboratory personnel. The etiologic factors for mosaic trisomy 18 are unknown, but advanced maternal age is an important factor that increases the risk of chromosomal nondisjunction in offspring. Nevertheless, the association between paternal age (40 years of age or older) and chromosomal abnormalities may be investigated [14,15].

We also found a *de novo* 25.72 Mb microduplication at Xp22.33p21.3, involving 109 genes. However, only 8 OMIM morbid genes have been described in association with intellectual disability, namely: *NLGN4X, AP1S2, NHS, CDKL5, RPS6KA3, MBTPS2, SMS, ARX*. Interstitial duplications of the short arm of the X chromosome have been rarely described. Female carriers of partial Xp duplication exhibit variable developmental defects, because of random or selective inactivation of X chromosome [16].

The encoded protein NLGN4X belongs to a family of neuronal cell surface proteins and mutations in this gene has been associated with autism and Asperger’s Syndrome (OMIM 300497). Pathogenic mutations in the X-linked *NLGN4X* in autism spectrum disorders and/or mental retardation are rare [16].

Moreover, the encode protein AP1S2 is a member of the adaptin protein family, disruption of the AP1 complex via mutations in AP1S2 could disrupt normal neurotransmitter processing within the synapse [17].

The encoded protein NHS may function during the development of the eyes, teeth and brain. Mutations in the *NHS* gene are related to Nance-Horan’s Syndrome (OMIM 302350), which is characterized by bilateral congenital cataracts, dental anomalies, craniofacial abnormalities and, in some cases, mental retardation. It is important in the limbic system, given the range of neuropsychological abnormalities, including mental retardation, autism, aggression, anxiety, stereotypical behavior and mood disturbance [18].

The *CDKL5* gene is a member of Ser/Thr kinase family and encodes a phosphorylated protein with a kinase activity. According to some researchers, female with *CDKL5* mutations experience no regression and the delay of psychomotor development is present since birth [19]. Alterations involving *CDKL5* have also been found in some patients with Hanefeld variants, a congenital form of Rett Syndrome, an X-linked dominant severe neurodevelopmental disorder that affect almost exclusively girls. Moreover, mutation or chromosomal translocations involving *CDKL5* have also been identified in patients with infantile spasms associated with mental retardation and in West’s Syndrome (OMIM 308350) patients [20].

The *RPS6KA3* gene encodes a member of the RSK (ribosomal S6 kinase) family of Ser/Thr kinases. The activity of this protein has been implicated in controlling cell growth and differentiation. Mutations in the *RPS6KA3* gene were first reported in Coffin-Lowry Syndrome (OMIM 303600) [21], and this gene also affects nonsyndromic X-linked intellectual disability and nonsyndromic X-linked intellectual disability without bone abnormalities [22]. In addition, one case was reported in which a boy with mild ID and a maternally inherited microduplication at Xp22.12. The duplicated region included *RPS6KA3*, a key gene related to mental retardation or intellectual disability in humans [21].

The *MBTPS2* gene encodes a intramembrane zinc metalloprotease, which is essential in development, and mutation in this gene can cause the Ichthyosis Follicularis, Atrichia, and Photophobia Syndrome, with or without BRESHECK Syndrome (OMIM 308205), which is an X-linked multiple congenital anomaly disorder with variable severity, and some patients have additional features, including mental retardation [23].

## Conclusion

This is the first case of a Brazilian child reported with complex genomic alterations involving chromosomes 7, 18 and X that went undetected by banding cytogenetics. However, the complex rearrangements were detected by chromosomal microarray analysis using clinical relevant probes. Synergistic effects from the rare genomic imbalances is likely responsible for the severe observed clinical phenotype in the proband. Further studies are required to define the role of the genes presented in this report to define the physiopathology of phenotypes similar to the one described here.

The rare and complex phenotypes need to be investigated to define the subsets and allow the phenotypes classification. Furthermore, this will allow adequate clinical management and a better follow up of the proband and the family.

### Materials and methods

Following the Pontifícia Universidade Católica de Goiás ethics approval (CAAE 0051.0.168.000-11) an informed consent was signed and peripheral blood was obtained for cytogenetic studies including karyotyping and CMA. Conventional cell culture, harvesting, and GTG banding followed standardized procedures [24]. Chromosome analyses were done using the software IKAROS® (Metasystems Corporation, Germany).

After karyotyping, the array analysis were performed on both patient and her parents, in order to determine the origin of potential DNA imbalances, either *de novo* or inherited. Genomic DNA was isolated from whole blood using QIAamp® DNA Mini kit (Qiagen, Germany). Total DNA (250 ng) was amplified, labeled, and hybridized using GeneChip CytoScan^TM^ HD array protocols (Affymetrix, USA) according to the manufacturer’s instructions. The array was designed specifically for cytogenetic research, including ≈ 2,696,550 CNV markers, 743,304 SNP markers, and > 1,953,246 non-polymorphic markers. CEL files obtained by scanning the arrays were analyzed using the Chromosome Analysis Suite (ChAS) software (Affymetrix, USA). Gains and losses that affected a minimum of 50 and 25 markers, respectively, in a 100 kb length were initially considered.

CNVs were classified according to their nature, based on [25,26]. In summary, the CNVs found in each patient and their biological parents were compared with genomic variants in public databases, including Database of Genomic Variants (DGV), Database of Chromosomal Imbalance and Phenotype in Humans using Ensemble Resources (DECIPHER), and CytoScan™ HD Array Database. CNVs were classified as pathogenic, likely pathogenic, and of unknown clinical significance, according to [24,25]. CMA analysis followed the manufacter’s instruction regarding mosaic, which follows the same principle of a complete CNV, a mosaic call was made by the software and included a clear result in SmoothSignal and Allele Peaks.

## Consent

Written informed consent was obtained from the parents of the patients for publication of this report and the accompanying images. A copy of the written consent is available for review by the Editor-in-Chief of this journal.

## Abbreviations

CMA: Chromosomal microarray analysis; CNV: Copy number variation; ID: Intellectual disability; ChAS: Chromosome analysis suite; ASD: Autism spectrum disorders.

## Competing interests

The authors declare that they have no competing interests.

## Authors’ contributions

IPP, LBM, ASC, DMS, CCS and ADC have made substantial contributions to conception and design, acquisition of data, analysis and interpretation of data; DMCC and CLR carried out the cytogenetics studies; IPP, AVM and RR carried out the chromosomal microarray analysis. All authors read and approved the final manuscript.
